# Maternal gut dysbiosis is associated with altered enteric and cortical inhibitory circuit development

**DOI:** 10.3389/fnana.2026.1801873

**Published:** 2026-04-10

**Authors:** Mohammed M. Nakhal, Faheema Nafees, Ayishal B. Mydeen, Abdulaziz Al Ali, Sarah Baloch, Rawdha Alkhalaf, Hasan M. Albalas, Ghassan M. Albalas, Gadayer Alharbi, Yauhen Statsenko, Nour al-dain Marzouka, Mohammad I. K. Hamad

**Affiliations:** 1 Department of Anatomy, College of Medicine and Health Sciences, United Arab Emirates University, Al Ain, United Arab Emirates; 2 Department of Radiology, College of Medicine and Health Sciences, United Arab Emirates University, Al Ain, United Arab Emirates; 3 Department of Genetics and Genomics, College of Medicine and Health Sciences, United Arab Emirates University, Al Ain, United Arab Emirates

**Keywords:** cortical interneurons, enteric nervous system, gut–brain axis, maternal dysbiosis, neurodevelopmental vulnerability, vertical microbial transmission

## Abstract

**Introduction:**

Maternal environmental factors critically influence neural circuit maturation during early development. The maternal gut microbiota has emerged as an important upstream regulator of offspring neurodevelopment, yet its role in shaping the structural organization of enteric and cortical inhibitory circuits remains poorly defined. Here, we examined whether gestational disruption of the maternal gut microbiota is associated with alterations in parallel enteric and cortical inhibitory circuit development.

**Methods:**

Maternal gut dysbiosis was induced in pregnant GAD67-GFP mice by oral vancomycin administration during gestation. Maternal and offspring microbiota were analyzed using full-length 16S rRNA gene sequencing to assess microbial diversity and vertical transmission. Offspring were examined at postnatal day 14 for intestinal morphology, altered barrier integrity, and enteric nervous system (ENS) organization. Cortical inhibitory circuits were analyzed by quantifying GAD67-positive interneuron density and performing three-dimensional morphological reconstruction in layers II/III of the somatosensory cortex, motor cortex, medial entorhinal cortex, and CA1 region of the hippocampus.

**Results:**

Maternal dysbiosis significantly reduced microbial diversity and disrupted maternal–offspring microbial transmission. These changes were associated with impaired intestinal development, including reduced crypt height, thinning of the muscularis propria, fragmented Claudin-1 expression, and reduced Auerbach’s plexus area without changes in neuronal density, indicating altered enteric network organization. In the brain, maternal dysbiosis induced region-specific cortical vulnerability, with reduced dendritic length and branching of GAD67-positive interneurons in the somatosensory and motor cortices, while interneuron morphology in the medial entorhinal cortex and hippocampus was preserved. Interneuron density was selectively reduced in the motor cortex.

**Discussion:**

These findings indicate that gestational maternal dysbiosis is associated with co-occurring structural alterations in intestinal and cortical inhibitory systems, selectively affecting inhibitory circuit architecture in sensorimotor regions. While the present model does not isolate microbiota-specific mechanisms from potential antibiotic-induced maternal physiological changes, the data support an association between disrupted maternal microbial ecology and offspring enteric and cortical neuroanatomical development during early postnatal life. These findings should be interpreted as descriptive associations and do not establish mechanistic gut–brain interactions.

## 1 Introduction

The maternal gut microbiome represents a dynamic and intergenerational ecosystem that profoundly shapes offspring development. Far beyond nutrient metabolism, maternal microbes influence fetal and neonatal physiology through metabolic, immune, and neuroendocrine pathways (Vuong et al., 2020; Alkuwaiti et al., 2025; Yassin et al., 2025a). During pregnancy, the gut microbiota undergoes selective remodeling characterized by reduced diversity and enrichment of immune-tolerant and energy-harvesting taxa (Koren et al., 2012). These changes ensure adequate nutrient mobilization and immune modulation but also establish the microbial foundation from which offspring colonization originates (Kimura et al., 2020). Vertical microbial transmission constitutes the cornerstone of early-life microbial assembly, facilitating the transfer of maternal microbes to the newborn through multiple complementary routes, vaginal delivery, breastfeeding, skin contact, and the perinatal environment (Ferretti et al., 2018; Shao et al., 2019). Vaginally delivered neonates acquire pioneer taxa such as *Lactobacillus*, *Prevotella*, and *Sneathia*, which initiate colonization of mucosal niches and shape early immune tolerance (Jašareviæ et al., 2021). During lactation, microbial transfer continues through the entero-mammary pathway, where immune cells traffic commensal bacteria such as *Bifidobacterium longum* and *Lactobacillus rhamnosus* into breast milk (Moossavi et al., 2019). Breast milk also provides oligosaccharides and secretory immunoglobulins that favor the growth of beneficial anaerobes and protect against opportunistic pathogens (Jeurink et al., 2013). In rodents, additional transmission occurs through coprophagy and nest exploration, allowing pups to ingest maternal anaerobic microbes that accelerate ecological succession and immune priming (Xiao and Zhao, 2023). Together, these processes generate a highly specific maternal-to-infant microbial continuum, ensuring stable microbial inheritance across generations (Korpela, 2021). When this transmission is perturbed, by cesarean section, formula feeding, or antibiotic exposure, offspring microbiota shift toward environmental taxa with diminished metabolic diversity and immune-regulatory capacity (Reyman et al., 2019). Such deviations can permanently alter intestinal physiology and neuroimmune development, increasing susceptibility to inflammation (Vatanen et al., 2018).

Vertical microbial transmission continues postnatally through maternal skin and oral contact, where commensals such as *Staphylococcus epidermidis* and *Corynebacterium kroppenstedtii* colonize the neonate during nursing and handling (Zeng et al., 2025). In rodents, coprophagy and nest exploration further facilitate ingestion of maternal anaerobes that accelerate gut colonization and immune education (Xiao and Zhao, 2023). These processes sustain a maternal–infant microbial continuum ensuring lineage-specific inheritance of key strains for immune and metabolic stability (Korpela et al., 2018). However, this fidelity is easily disrupted. Cesarean delivery, formula feeding, and perinatal antibiotic exposure redirect microbial seeding away from maternal *Bacteroides* and *Bifidobacterium* lineages toward environmental opportunists lacking immunoregulatory capacity (Reyman et al., 2019; Shao et al., 2019). Such dysbiotic inheritance compromises intestinal development and long-term host–microbe symbiosis, predisposing offspring to immune disorders (Vatanen et al., 2018). Antibiotic-induced dysbiosis during pregnancy further reduces microbial richness, depletes butyrate-producing taxa, and promotes expansion of *Gammaproteobacteria* (Mydeen et al., 2025; Yassin et al., 2025b). Human studies link perinatal antibiotic use to delayed microbial maturation and increased allergic, metabolic, and cognitive dysfunctions (Vatanen et al., 2018; Shao et al., 2019). Collectively, these findings indicate that maternal dysbiosis undermines vertical microbial fidelity, introducing ecological instability that represents a modifiable risk factor for developmental disease (Grech et al., 2021).

The microbiota–gut–brain axis (MGBA) provides a biochemical and neural framework through which gut microbes influence central nervous system (CNS) development (Cryan et al., 2019; Nakhal et al., 2024; Yassin et al., 2025b). Microbial metabolites such as short-chain fatty acids (SCFAs), tryptophan derivatives, and secondary bile acids act as systemic signaling molecules that regulate neurogenesis, microglial activation, and synaptic remodeling (Strandwitz, 2018). Butyrate, produced by *Roseburia* and *Faecalibacterium*, enhances blood–brain barrier integrity and increases brain-derived neurotrophic factor (BDNF) expression, promoting dendritic growth and synaptic plasticity (Silva et al., 2020). Conversely, loss of these metabolites through dysbiosis induces systemic inflammation, and elevates intestinal permeability (Bonaz et al., 2018). Germ-free and antibiotic-treated animals display impaired cortical interneuron maturation, reduced dendritic arborization, and behavioral phenotypes consistent with anxiety and cognitive dysfunction (Heijtz et al., 2011; Nakhal et al., 2025a). These findings underscore that maternal microbial stability is critical for shaping offspring brain architecture through both metabolic and immune mechanisms. The ENS represents a key anatomical and functional component of the gut–brain axis, integrating microbial, immune, and metabolic signals. Organized into the myenteric (Auerbach’s) and submucosal (Meissner’s) plexuses, the ENS regulates intestinal motility, secretion, and epithelial barrier maintenance (Furness, 2012). Gut microbes regulate ENS development by providing trophic metabolites such as SCFAs and by modulating neurogenesis and synaptic plasticity (Obata et al., 2020). Although the neurological effects of adult dysbiosis are well recognized, its intergenerational impact remains unclear. In particular, how disrupted vertical microbial transmission influences enteric and cortical neuronal development has not been defined. Here, we used a gestational vancomycin model of maternal dysbiosis in GAD67-GFP mice to examine how microbial imbalance during pregnancy affects offspring gut morphology, ENS organization, and cortical inhibitory network maturation. Specifically, we evaluated changes in microbial diversity and vertical transmission fidelity, alterations in intestinal barrier integrity and ENS structure, and region-specific effects on cortical GABAergic interneuron morphology and density. We examined whether maternal dysbiosis is associated with alterations in enteric and cortical development. This framework contextualizes maternal microbiota imbalance in relation to enteric and cortical developmental changes and highlights vertical transmission as a critical determinant of offspring vulnerability to gastrointestinal and neurodevelopmental disorders.

## 2 Materials and methods

### 2.1 Animals and experimental design

**FIGURE 1:**
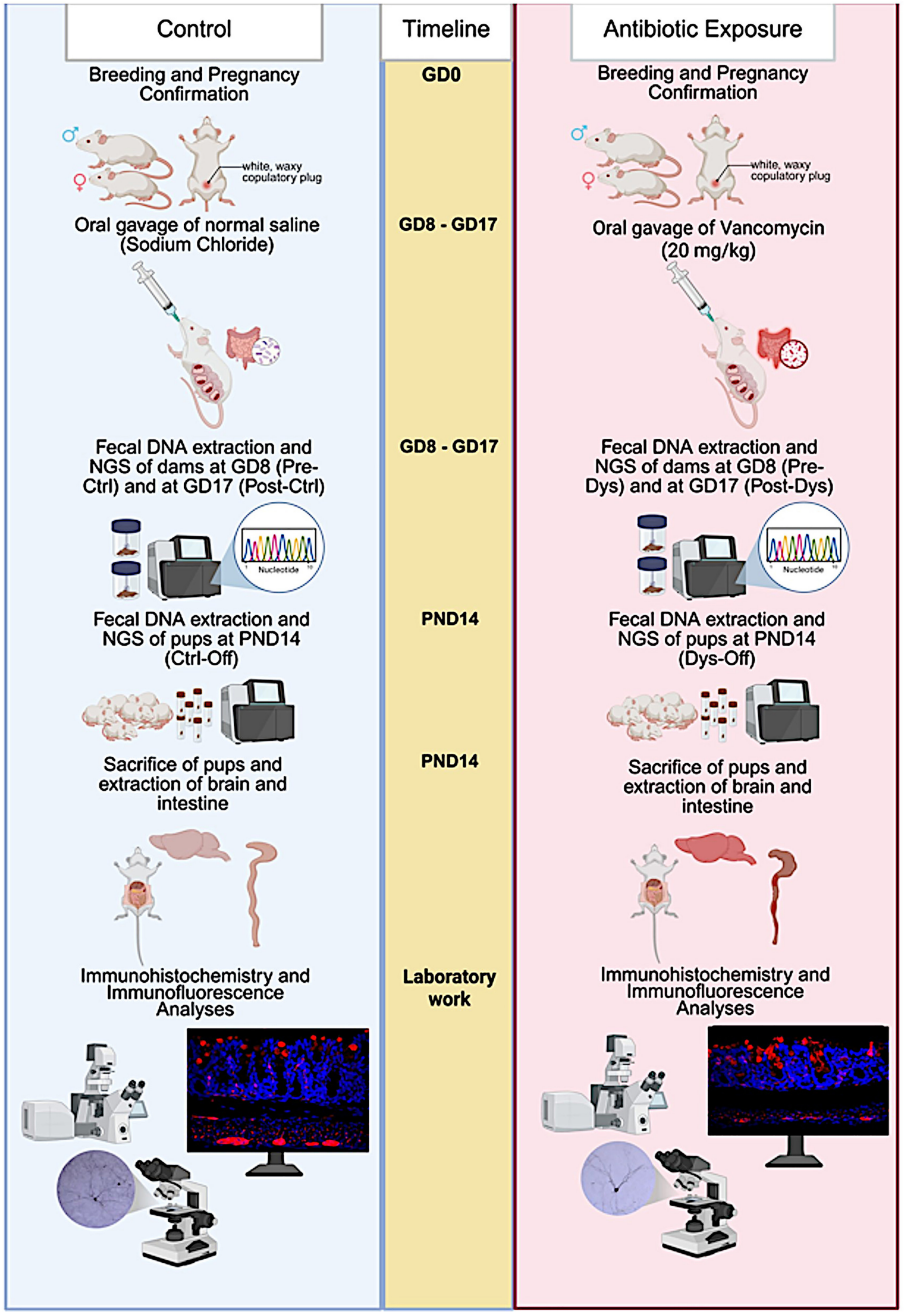
Experimental design outlining antibiotic-induced maternal dysbiosis and vertical transmission timeline. Pregnant dams were confirmed at gestational day 0 (GD0) by detection of the white, waxy copulatory plug following mating then separated. From GD8 to GD17, control dams received oral gavage of normal saline, while antibiotic-exposed dams received vancomycin (20 mg/kg) to induce gestational dysbiosis. Fecal samples were collected from dams at GD8 (Pre-Ctrl and Pre-Dys) and GD17 (Post-Ctrl and Post-Dys) for 16S rRNA gene sequencing. At postnatal day 14 (PND14), fecal samples were collected from pups (Ctrl-Off and Dys-Off), followed by sacrifice and extraction of brain and intestinal tissues. Laboratory work included immunofluorescence analyses to assess intestinal barrier integrity, and interneurons morphology in the offspring. The study included 12 pregnant dams (control, *n* = 6; dysbiosis, *n* = 6) and 24 offspring (control, *n* = 12; dysbiosis, *n* = 12).

Experiments were carried out using GAD67-EGFP transgenic mice (3-month-old pregnant females) and their postnatal day (PND) 14 offspring. Postnatal day 14 was selected because it corresponds to a critical window of rapid dendritic growth, synaptogenesis, and inhibitory circuit refinement in the murine cortex, as well as ongoing maturation of enteric neural networks. All animals were maintained under controlled environmental parameters, temperature was maintained at 22 ± 1 °C, relative humidity between 50 and 60%, and a 12-h light/dark cycle. Mice had unrestricted access to standard chow and water throughout the study. Following an acclimation period, female mice were paired overnight with males, and the appearance of a white, waxy copulatory plug at the vaginal opening was considered gestational day 0 (GD0). Pregnant females were then randomly allocated into two experimental groups: a control (*n* = 6) and a maternal dysbiosis group (*n* = 6). Maternal gut dysbiosis was induced by administering vancomycin (20 mg/kg/day, oral route) dissolved in sterile water from GD8 to day GD17, as described in previous studies (Nakhal et al., 2025a). Control animals received the vehicle (sterile water) for the same duration. Fecal samples were obtained from each control dam prior to vehicle administration (Pre-Ctrl) and following treatment at GD 17 (Post-Ctrl), as well as from antibiotic-treated dams before treatment initiation (Pre-Dys) and after vancomycin exposure (Post-Dys), for subsequent microbiome analysis (Figure 1). All procedures were conducted in compliance with institutional animal care and use guidelines and adhered to established ethical standards for animal research. Furthermore, detailed descriptions of the research design, experimental procedures, and analytical methods are provided in accordance with the ARRIVE Guidelines 2.0 to ensure transparency and reproducibility.

### 2.2 Fecal DNA extraction and 16S rRNA gene sequencing

During the experimental period, animals were single housed to enable accurate tracking of individual fecal sampling and microbiome sequencing. Genomic DNA was isolated from fecal samples using the QIAamp PowerFecal Pro DNA Kit (Qiagen, Hilden, Germany) following the manufacturer’s protocol under aseptic conditions. The concentration and purity of the extracted DNA were measured with a Qubit 4 fluorometer (Thermo Fisher Scientific, United States) employing the double-stranded DNA High-Sensitivity (dsDNA HS) assay kit. Amplification of the full-length 16S rRNA gene (V1–V9 region, approximately 1.5 kb) was performed using the 16S Barcoding Kit (SQK-16S114.24, Oxford Nanopore Technologies, United Kingdom) in combination with the LongAmp Hot Start Taq 2 × Master Mix (New England Biolabs, United States). PCR amplification was carried out following the manufacturer’s cycling parameters. Following amplification, the PCR products were quantified, pooled in equimolar concentrations, and purified using AMPure XP magnetic beads (Beckman Coulter, United States). Library preparation was conducted using 50 fmol of pooled DNA, and sequencing was performed on a MinION Mk1C platform (Oxford Nanopore Technologies, United Kingdom) equipped with FLO-MIN114 flow cells for approximately 48 h (Figure 1).

### 2.3 Microbiome sequencing and data analysis

Raw sequencing reads were processed using MinKNOW software (version 6.0.14, Oxford Nanopore Technologies) for real-time base calling, applying a minimum quality filter of Q9. Taxonomic classification of FASTQ files was performed with Kraken2 using the SILVA reference database, and the resulting taxonomic tables were exported via Epi2me (Oxford Nanopore Technologies) in TXT format for downstream analyses. Low-abundance features were excluded using a minimum threshold of four read counts and 10% prevalence across samples. Normalization of the filtered data was conducted by total sum scaling (TSS) within MicrobiomeAnalyst 2.0 (McGill University, Canada). Alpha diversity metrics, including Chao1, Shannon, and Simpson indices, were calculated to estimate species richness, evenness, and dominance, respectively. Differences among groups were analyzed using non-parametric Mann–Whitney U or Kruskal–Wallis tests, with Benjamini–Hochberg correction applied for multiple comparisons. Beta diversity was assessed using Bray–Curtis dissimilarity indices and visualized through Principal Coordinates Analysis (PCoA). Statistical differences in community composition were further evaluated by permutational multivariate analysis of variance (PERMANOVA). To assess maternal–offspring microbial similarity, a multifactor linear model was constructed at the species level, enabling identification of conserved taxa and compositional relationships between dams and their offspring. Differential abundance analyses were carried out using Linear Discriminant Analysis Effect Size (LEfSe), employing a log10 LDA score threshold of 2 to identify discriminative taxa. Relative abundance boxplots, and heatmaps were generated using SigmaPlot (Systat Software, Inc.), while ordination and other statistical workflows were completed in MicrobiomeAnalyst. Statistical significance was defined as *p* < 0.05.

### 2.4 Swiss-roll preparation of intestinal tissue

Rodents were euthanized via cervical dislocation, and the entire gastrointestinal tract, including the small and large intestines, were immediately removed and moistened with 1× phosphate-buffered saline (PBS). The ileum was dissected from the small intestine, divided into smaller segments, and positioned with the caudal end facing the investigator. To remove residual fecal matter, ice-cold PBS was gently flushed through the intestinal lumen from the caudal end. After initial cleaning, tissues were immersed in fresh PBS, slit longitudinally, and rinsed again to ensure complete clearance of luminal contents. To enable visualization of an extensive section of intestinal architecture within a single histological plane, the Swiss-roll technique was employed (Moolenbeek and Ruitenberg, 1981). Cleaned segments were tightly rolled around a thin wooden stick with the mucosal surface facing outward, secured with stainless steel pins, and fixed overnight in Bouin’s solution. Following fixation, tissues underwent dehydration began with 70% ethanol at 4 °C overnight. The next day, samples were brought to room temperature and transferred to 90% ethanol for 1 h, followed by two consecutive incubations in 100% ethanol (2 h each). Clearing was performed using a 1:1 xylene–ethanol mixture for 30 min, then twice in xylene alone for an additional 30 min per step. Paraffin infiltration was carried out in three 30-min cycles, after which the tissue rolls were embedded in paraffin using a Thermo Scientific HistoStar workstation and stored at -8 °C. Sections were cut at 5 μm thickness using a Leica RM 2125 RTS microtome (Heidelberg, Germany) and mounted onto 0.5% gelatin-coated slides for subsequent histological and immunofluorescence analysis.

### 2.5 Animal transcardial perfusion and immunohistochemistry

By day 16 of the experiment, sufficient time had elapsed for microbial alterations to potentially influence neural morphology, particularly aspects such as interneuron density and dendritic structure. This time point was therefore selected to evaluate the morphological effects of microbiota modulation in the mature brain, providing insights into how gut microbial shifts may alter pre-established neuronal networks. Animals were deeply anesthetized with 5% isoflurane and transcardially perfused with phosphate-buffered saline (PBS), followed by 4% paraformaldehyde (PFA) prepared in 0.1 M phosphate buffer (pH 7.2). After perfusion, brains were carefully removed and postfixed overnight in the same fixative. The tissue was then cryoprotected in 30% sucrose (in PBS, pH 7.4) for an additional 24 h. Using a vibratome, 150-μm parasagittal brain sections were prepared from both hemispheres, encompassing the lateral entorhinal cortex and extending to the midline. The Allen Brain Atlas was used as a reference to accurately delineate and collect slices from the mEC, Hp, SSC, and MC. For enhanced visualization and structural analysis, sections underwent immunohistochemical processing with anti-EGFP antibody to convert the fluorescent signal into a stable diaminobenzidine (DAB) reaction product, facilitating high-resolution three-dimensional neuronal reconstruction. Brain sections were washed repeatedly in Tris-buffered saline (TBS; 50 mM Tris, 150 mM NaCl, pH 7.6) and permeabilized with TBS containing 0.1% Triton X-100 (TBST). To minimize non-specific binding, slices were blocked for 1 h in 1% normal goat serum prepared in TBST. Subsequently, they were incubated for 24 h at room temperature with the primary antibody: chicken anti-GFP (1:8,000, Abcam; ab13970). After two washes in TBS, sections were treated with the biotinylated rabbit anti-chicken secondary antibody (1:300; Dako, Cat# E043201-8) for 3 h at room temperature. Finally, immunolabeling was visualized using the avidin–biotin complex (ABC) horseradish peroxidase method, with diaminobenzidine (DAB) serving as the chromogenic substrate.

### 2.6 GAD67-positive interneuron quantification

Quantitative assessment of GAD67-expressing interneurons was performed using 150-μm parasagittal serial brain sections. Parasagittal sections were selected to permit simultaneous and consistent visualization of medial (mEC, hippocampus) and lateral (SSC, MC) cortical regions within the same anatomical plane. Sections were collected at defined mediolateral coordinates relative to the midline, and anatomical boundaries were identified according to the Allen Brain Atlas to ensure regional consistency across animals. For cortical regions (SSC, MC, and mEC), interneuron density was quantified using a standardized laminar sampling strategy. For each region and each section, three areas of interest (AOIs) were systematically selected from layers II/III. The density values obtained from these three AOIs were averaged to generate a single representative value per region per section, thereby ensuring sampling across the full cortical thickness. For the hippocampus, density quantification was restricted to the CA1 subregion as defined by the Allen Brain Atlas. AOIs were selected within the anatomical boundaries of CA1 without separate subdivision of individual CA1 layers (stratum oriens, pyramidale, radiatum, and lacunosum-moleculare). Thus, reported values represent averaged interneuron density within the CA1 territory. High-resolution images of neuronal somata were acquired using a Zeiss light microscope (Germany) equipped with a 40 × objective lens and a calibrated counting grid to ensure standardized sampling area. EGFP-immunolabeled cells were identified using the anti-EGFP antibody. All captured images were analyzed using MacBiophotonics imaging software for quantification. For each animal, values from multiple sections were averaged prior to statistical analysis, and the animal was considered the biological unit for comparison.

### 2.7 Immunofluorescence

Tissue sections were dewaxed in xylene for 5 min each and rehydrated through graded ethanol (absolute ethanol, 3 min twice; 95 and 70% ethanol, 3 min each), followed by rinsing in phosphate-buffered saline (PBS) and distilled water. Antigen retrieval was performed using citrate buffer (pH 6.0) under controlled heating conditions. After three washes in PBS (5 min each), slides were incubated with primary antibodies overnight at 4 °C. The following primary antibodies were used: anti-PGP9.5 (Rabbit, HUABIO; 1:400) to visualize enteric neurons and anti-Claudin-1 (Rabbit, Proteintech; 1:350) to assess epithelial tight-junction integrity. On the following day, sections were washed in PBS and incubated for 1 h at room temperature with the corresponding secondary antibodies: goat anti-rabbit Alexa Fluor^®^ 594 (1:300, Invitrogen A11012) for fluorescence detection. After final PBS washes, slides were mounted with a DAPI-containing medium (Abcam AB104139) to counterstain nuclei. Images were captured using an Olympus confocal microscope at 40 × magnification, and fluorescence intensity and structural organization of PGP9.5- and Claudin-1-positive regions were quantified using ImageJ software, following standardized image-analysis protocols.

### 2.8 Three-dimensional neuron reconstruction of interneurons

EGFP-immunolabeled interneurons were reconstructed in three dimensions using the Neurolucida system (MicroBrightField, United States) under 1,000 × magnification. Morphological quantification of GAD67-positive interneurons included measurements of mean dendritic length, mean number of dendritic segments, and the number of primary dendrites per neuron as described previously (Hamad et al., 2021; Nakhal et al., 2025b). For cortical regions (SSC and MC), interneurons selected for three-dimensional reconstruction were exclusively sampled from layers II/III to ensure laminar consistency across animals and experimental groups. Neurons were selected based on clear visualization of the complete dendritic arbor and somatic localization within layers II/III, as defined according to the Allen Brain Atlas. Care was taken to avoid inclusion of interneurons from deeper cortical layers to prevent laminar heterogeneity in morphological comparisons. Interneurons selected for three-dimensional reconstruction were chosen based on clear morphological isolation from neighboring GFP-positive cells to avoid overlap artifacts inherent to dense reporter lines. Cells located in close proximity to other labeled interneurons were excluded from reconstruction analysis to ensure accurate single-cell tracing. To minimize subtype composition bias, interneurons were selected randomly within layers II/III without preselection based on morphological class, and reconstruction and analysis were performed blind to treatment group. To evaluate dendritic branching complexity, Sholl analysis was conducted by counting dendritic intersections at 10 μm concentric intervals radiating from the soma (Sholl, 1953; Hamad et al., 2014). This analysis allowed the identification of regions where dendritic arborization differed significantly between groups.

### 2.9 Statistical analysis for morphological data

Statistical analyses were performed using SigmaStat 12 (SPSS Inc., United States). Comparisons between two groups were conducted using either a Student’s unpaired t-test or a Mann–Whitney U test, depending on the results of the Shapiro–Wilk normality test. When more than two groups were compared, a one-way ANOVA followed by a Holm–Sidak post hoc test was applied for normally distributed data. In cases where the normality assumption was not met, a one-way ANOVA on ranks with a Tukey *post-hoc* test was used to determine significant group differences. A value of *p* < 0.05 was considered statistically significant. For Sholl intersection analyses, a two-way repeated-measures ANOVA was applied with treatment (dysbiosis vs. control) as the between-subject factor and radial distance from the soma as the within-subject factor. Violations of sphericity were corrected using the Greenhouse–Geisser adjustment, and *post-hoc* comparisons at individual radial distances were conducted using Bonferroni-corrected *t*-tests to account for multiple testing. Associations between bacterial abundance and morphological parameters of the enteric nervous system and cortical interneurons were assessed using Pearson’s correlation. Bacterial read counts were log-transformed prior to analysis, and correlations were calculated using complete-case observations. Correlation matrices were generated and visualized in R (v4.3.1). No causal inferences were drawn from correlation analyses.

## 3 Results

### 3.1 Validation of the maternal dysbiosis model

**FIGURE 2:**
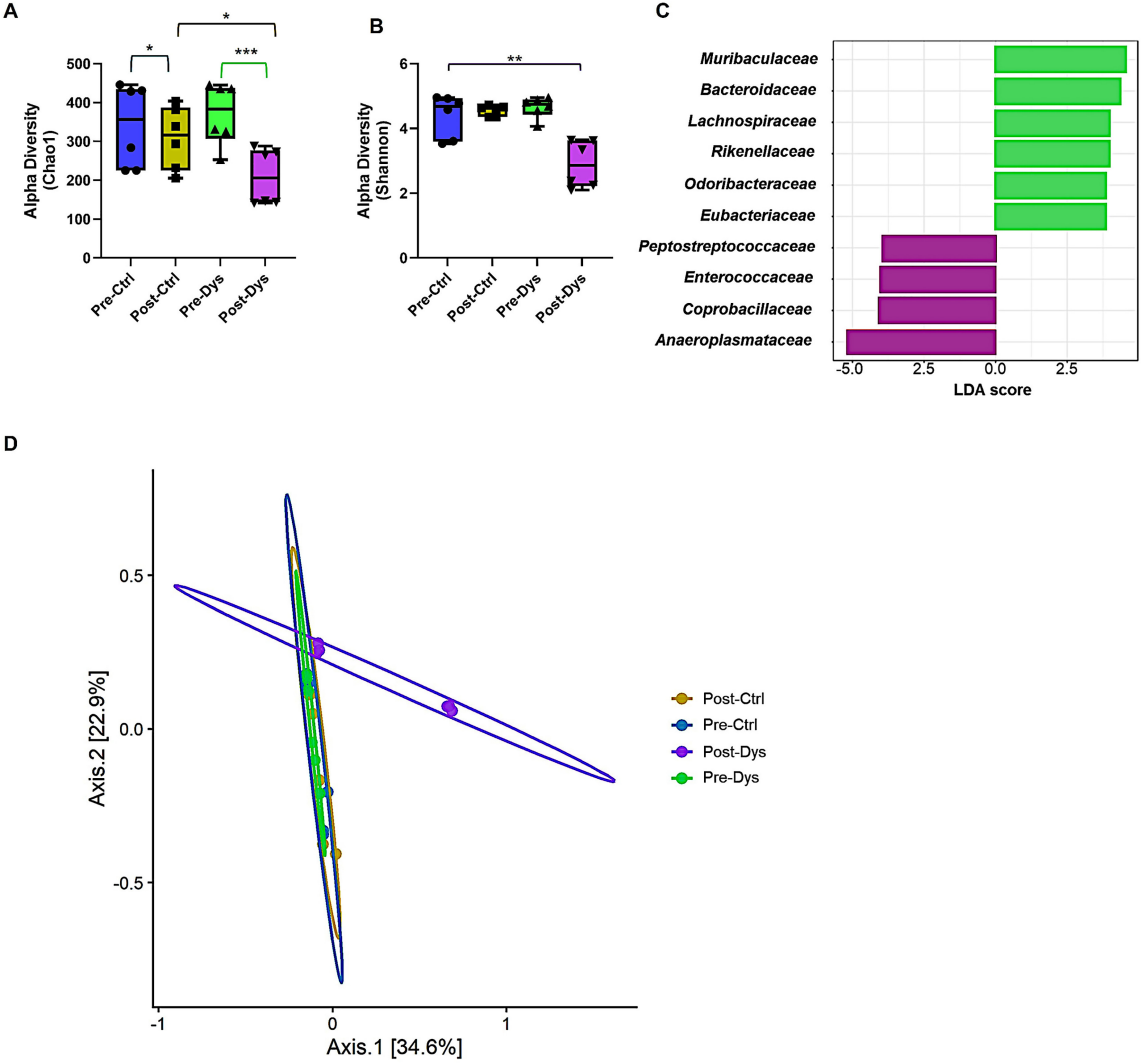
Antibiotic-induced maternal dysbiosis alters microbial diversity and composition. **(A)** Alpha diversity indices (Chao1: richness; Shannon: evenness + richness) showing significant post-treatment declines in vancomycin-exposed dams (Post-Dys) compared with pre-treatment (Pre-Dys) and control groups (Pre-/Post-Ctrl). Statistical analysis was performed using one-way ANOVA followed by *post hoc* multiple comparisons; *p < 0.05, **p < 0.01, ***p < 0.001. **(B)** Principal Coordinates Analysis (PCoA) of Bray–Curtis dissimilarities demonstrating distinct clustering of Post-Dys samples (purple) apart from all other groups. Significance confirmed by PERMANOVA (*p* = 0.00). **(C)** Linear Discriminant Analysis Effect Size (LEfSe) identifying bacterial taxa driving separation between groups. Control dams were enriched in beneficial commensals (green bars), while Post-Dys samples displayed enrichment of opportunistic pathobionts (purple bars). Microbiome analyses were performed on fecal samples from 12 pregnant dams (control, *n* = 6; dysbiosis, *n* = 6). **(D)** Principal coordinates analysis (PCoA) based on Bray–Curtis dissimilarities showing separation of Post-Dys samples from other groups. Microbiome profiling was performed on fecal samples from pregnant dams (control, *n* = 6; dysbiosis, *n* = 6).

To confirm successful induction of maternal gut dysbiosis, we longitudinally analyzed fecal microbiota from pregnant dams before (Pre-Dys) and after oral vancomycin treatment (Post-Dys) and compared with time-matched controls. Alpha diversity indices revealed marked reductions in microbial richness and evenness following antibiotic exposure. Specifically, the Chao1 index showed a significant decline in Post-Dys dams compared with all other groups (Figure 2A, *p* < 0.05–0.001), while control animals exhibited only a modest gestational decrease (Figure 2A). Similarly, the Shannon diversity index decreased sharply in Post-Dys dams (*p* < 0.01; Figure 2B), indicating a substantial loss of both taxa number and community balance, In contrast to the dysbiotic dams, the Shannon diversity index remained stable in the post-gestational control group (Post-Ctrl), indicating that normal pregnancy did not reduce microbial evenness. Linear Discriminant Analysis Effect Size (LEfSe) differential abundance analysis indicated distinct signatures of microbiota at family level between the groups (Figure 2C). Since families typically associated with beneficial commensals, including *Muribaculaceae, Bacteroidaceae, Lachnospiraceae, Rikenellaceae*, and *Odoribacteraceae* were enriched in control dams, whereas families known to include opportunistic or pathobiont taxa, such as *Peptostreptococcaceae, Enterococcaceae, Coprobacillaceae*, and *Anaeroplasmataceae*, thrived in the Post-Dys group. To further assess treatment-driven changes in community composition, beta diversity was examined using Bray–Curtis dissimilarities. Principal Coordinates Analysis (PCoA) demonstrated clear segregation of the Post-Dys group from all others along the primary axis (Axis 1 = 80.5 % variance explained). This separation was statistically supported by PERMANOVA (*F* = 8.71; *R*^2^ = 0.77; *p* = 0.007), confirming a pronounced and consistent microbiota shift following vancomycin administration (Figure 1D). Collectively, these data validate that vancomycin treatment induces a classical dysbiosis phenotype during pregnancy, marked by loss of diversity, collapse of community evenness, and expansion of opportunistic taxa, while control gestation exerts only mild compositional remodeling. This established the experimental foundation for assessing how maternal microbial perturbation influences offspring gut and brain development.

### 3.2 Maternal dysbiosis reduces microbial diversity and vertical transmission fidelity in offspring

**FIGURE 3:**
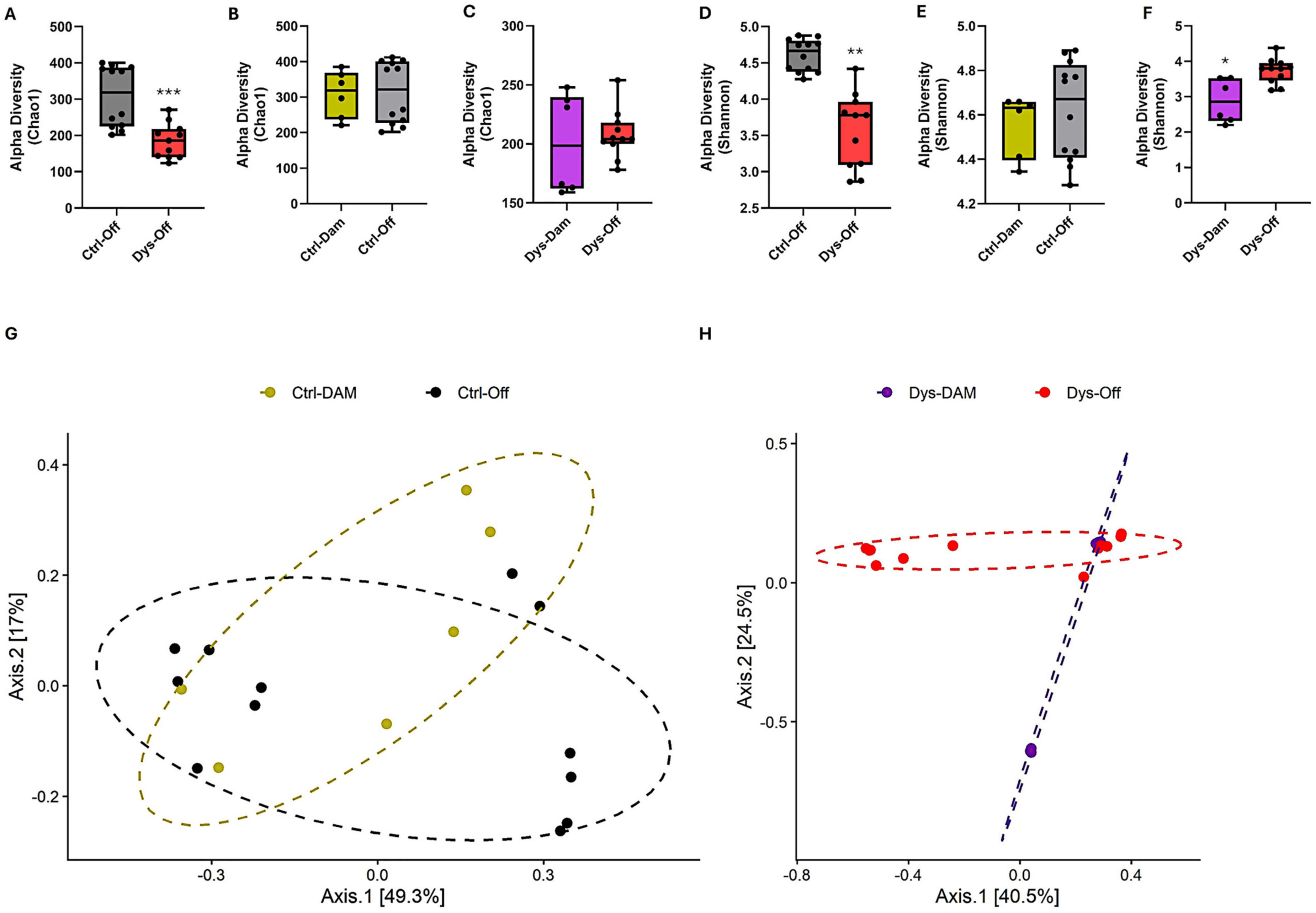
Maternal dysbiosis diminishes offspring microbial diversity and vertical transmission fidelity. **(A–F)** Alpha diversity indices (Chao1 and Shannon) for control and dysbiotic dams (Ctrl-Dam, Dys-Dam) and their offspring (Ctrl-Off, Dys-Off). Dysbiotic offspring show significantly reduced richness and evenness compared with controls (**A**,**D**) and closely mirror their dysbiotic mothers **(C,F)**. Control dyads exhibit stable diversity across generations **(B,E)**. Data represent mean ± SEM; statistical significance determined by one-way ANOVA with post hoc multiple comparisons; *p < 0.05, **p < 0.01, ***p < 0.001. **(G**,**H)** Principal Coordinates Analysis (PCoA) of Bray–Curtis dissimilarities illustrating distinct clustering of control and dysbiotic groups. **(G)** Comparison between control and dyads. **(H)** Comparison between dysbiotic dyads. Significance assessed by PERMANOVA (*p* = 0.015). Microbiome analyses were performed on fecal samples from 12 pregnant dams (control, *n* = 6; dysbiosis, *n* = 6) and their offspring (24 pups total; control, *n* = 12; dysbiosis, *n* = 12).

To determine whether antibiotic-induced maternal dysbiosis alters microbial inheritance in offspring, We compared the gut microbiota composition of control (Ctrl-Dam) and dysbiotic dams (Dys-Dam), along with their PND14 offspring (Ctrl-Off and Dys-Off). Both the Chao1 (richness) and Shannon (richness–evenness) indices showed significant decreases in Dys-Off compared with Ctrl-Off groups (*p* < 0.01–0.001; Figures 3A,D). Moreover, the Dys-Dam conveyed their reduced microbial richness to their offspring, as reflected by the significant decline in Chao1 index (Figure 3C), while a similar but non-significant trend was observed for microbial evenness (Figure 3F), indicating vertical inheritance of the dysbiotic signature. In contrast, offspring of control dams displayed comparable diversity to their mothers, with no significant intergenerational difference (Figures 3B,E). Beta diversity analysis further supported these patterns. PCoA of Bray–Curtis dissimilarities revealed distinct clustering of Dys-Dam and Dys-Off communities compared with their respective control counterparts (Figures 3G,H). PERMANOVA confirmed significant treatment-driven segregation (*F* = 18.22; *R*^2^ = 0.72; *p* = 0.015), reflecting a strong compositional impact of maternal antibiotic exposure on offspring microbial assembly. Together, these results demonstrate that maternal dysbiosis compromises vertical microbial transmission, leading to an intergenerational reduction in diversity and a lasting compositional divergence between control and dysbiotic offspring.

### 3.3 Covariate-adjusted regression analysis revealed disruption of vertical microbial transmission under dysbiosis

**FIGURE 4:**
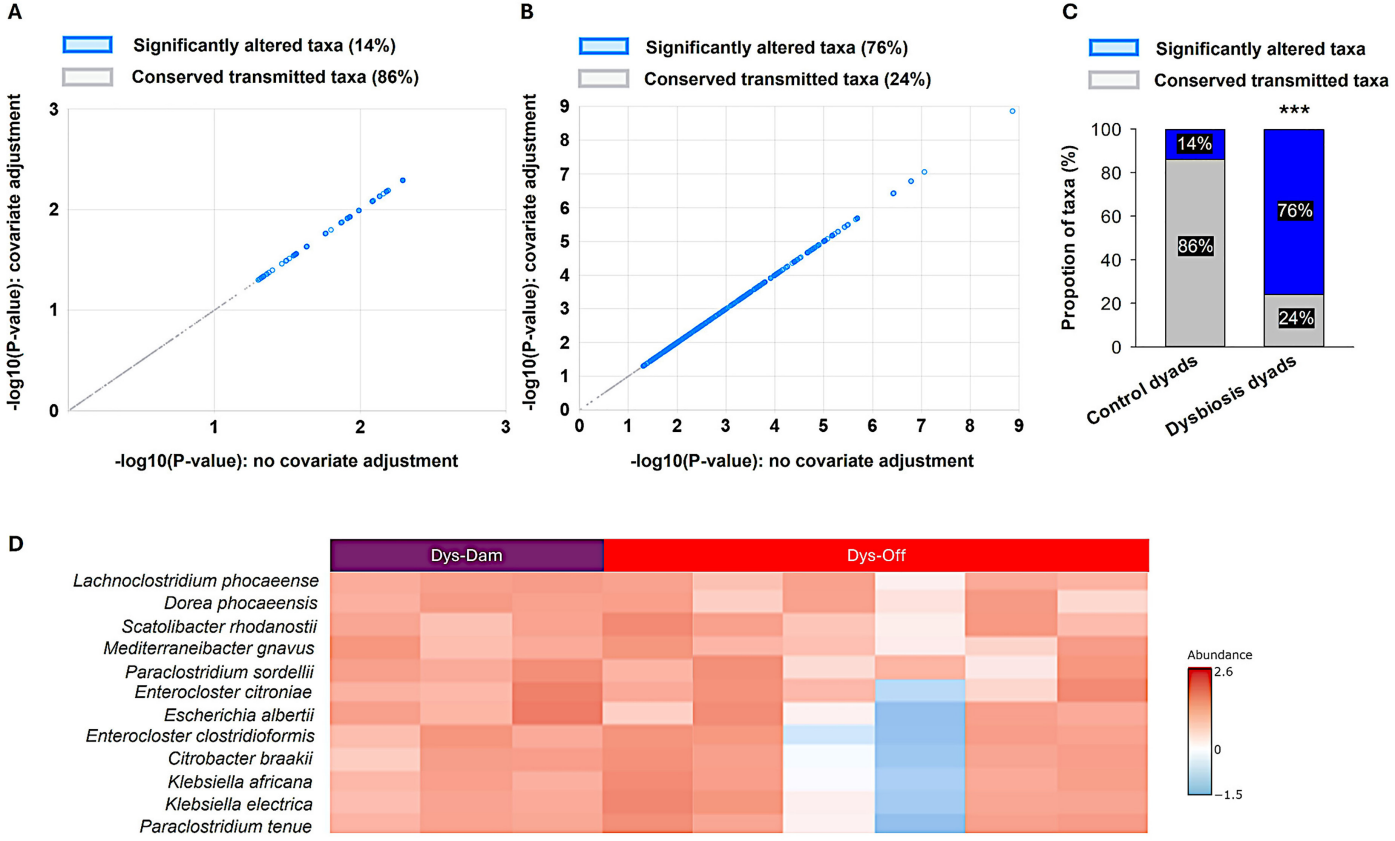
Maternal dysbiosis disrupts vertical microbial transmission between dams and offspring. **(A)** Multiple linear regression with covariate adjustment (MicrobiomeAnalyst) identified significantly altered taxa (blue, 14%) and conserved transmitted taxa (gray, 86%) between control dams and pups. **(B)** Covariate-adjusted multiple regression (MicrobiomeAnalyst) analysis in dysbiotic identified a profound shift, with 76% of taxa significantly altered and only 24% remaining conserved. **(C)** Comparative analysis of the proportions of significantly altered versus conserved taxa between control and dysbiotic dyads using a chi-square test (SigmaPlot) confirmed a significant disruption of vertical microbial transmission (***p < 0.001). **(D)** Heatmap of maternally transmitted pathobionts in dysbiotic dyads (MicrobiomeAnalyst). Each column represents an individual sample (dysbiotic dam, purple; dysbiotic offspring, red), and each row represents transmitted species. The colored scale indicates relative abundance (z-score normalized): red reflects higher abundance and blue indicates lower abundance relative to the dataset mean. Values near 0 denote average abundance across samples, while ± 2.5 represent the upper and lower extremes. Analyses were performed on 12 maternal–offspring dyads derived from 12 pregnant dams (control, *n* = 6; dysbiosis, *n* = 6) and their offspring (control, *n* = 12; dysbiosis, *n* = 12).

To measure maternal–offspring microbial inheritance fidelity, a multiple linear regression with covariate adjustment at species level was performed. In control dyads, majority of taxa (86%) remained conserved between dams and pups, with only 14% showing significant changes after adjustment (Figure 4A). Accordingly, this indicates a stable vertical transmission of the maternal microbiome during pregnancy, delivery, and breastfeeding. In contrast, dysbiotic dyads exhibited a marked loss of transmission fidelity (Figure 4B), with 76% of taxa significantly altered and only 24% retained across generations. Chi-square comparison between the two groups (Figure 4C) confirmed a significant reduction in the proportion of conserved taxa in the dysbiosis condition (*p* < 0.001). Heatmap profiling of the transmitted taxa of the dysbiotic dyads (Figure 4D) revealed that several opportunistic and pathobiont species including *Lachnoclostridium phocaeense, Dorea phocaeensis, Enterocloster clostridioformis*, and *Klebsiella Africana*, were selectively passed to the offspring and maintained elevated abundance levels, suggesting preferential vertical inheritance of dysbiosis-associated microbes. Together, these findings demonstrate that maternal gut dysbiosis not only diminishes microbial transmission stability but also biases inheritance toward potentially pro-inflammatory taxa.

### 3.4 Maternal dysbiosis compromises offspring gut barrier integrity and enteric neuronal architecture

**FIGURE 5:**
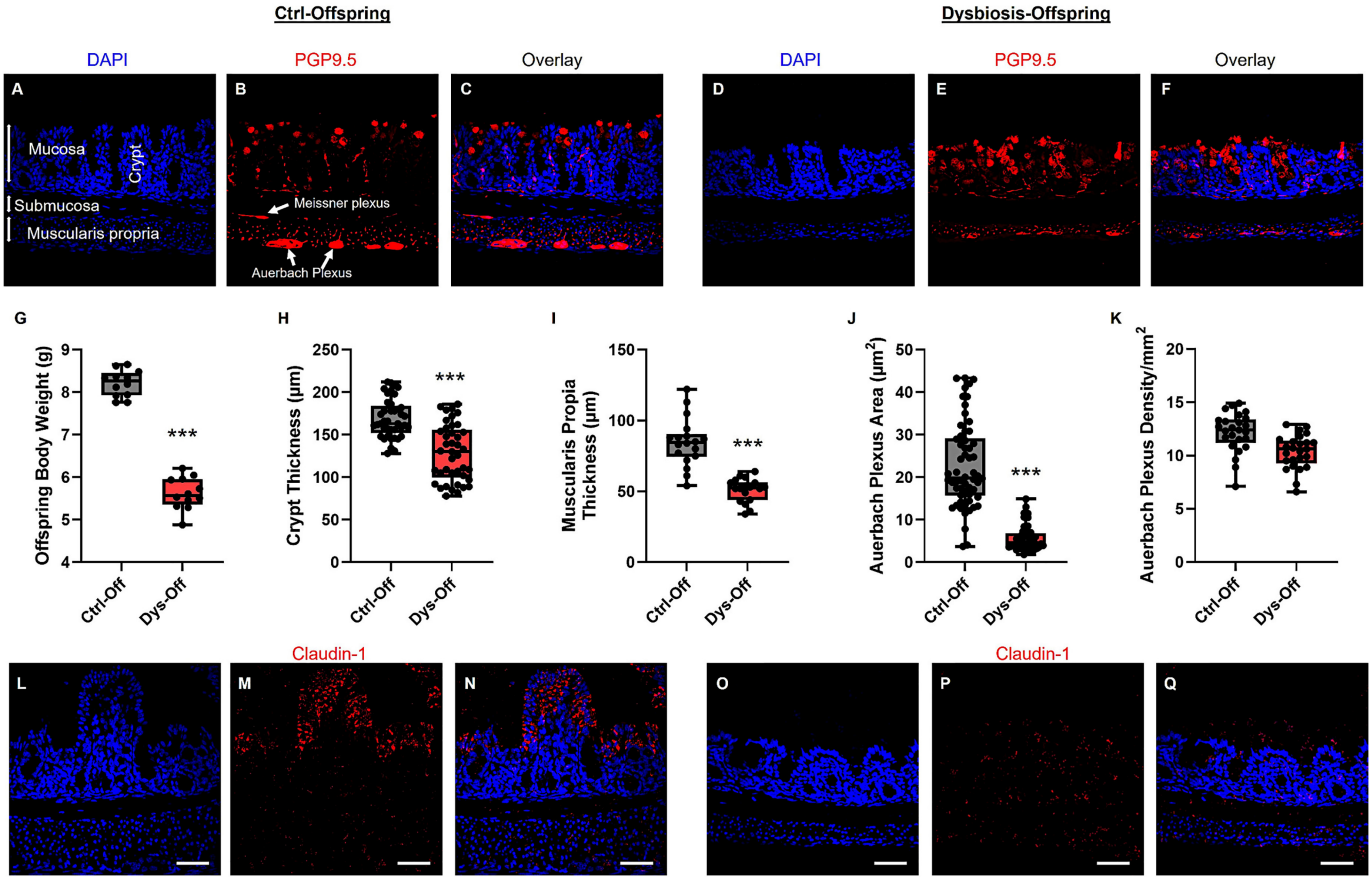
Maternal dysbiosis disrupts offspring gut morphology, altered barrier integrity, and enteric neuronal organization. **(A–F)** Representative confocal images of the large intestine from control offspring (Ctrl-Off) and offspring from dysbiotic dams (Dys-Off) immunostained for the pan-neuronal marker PGP9.5 (red) and counterstained with DAPI (blue). **(G–K)** Quantitative morphometric analyses showing reduced body weight **(G)**, crypt thickness **(H)**, muscularis propria thickness **(I)**, and Auerbach’s plexus area **(J)** in Dys-Off relative to Ctrl-Off. Auerbach’s plexus density **(K)** was not significantly altered. Data are presented as mean ± SEM; ***p < 0.001, unpaired *t*-test. **(L–Q)** Representative immunofluorescence images of intestinal sections stained for Claudin-1 (red) and DAPI (blue). Ctrl-Off samples exhibit continuous Claudin-1 labeling along the apical epithelial surface **(L–N)**. Dys-Off intestines display fragmented and discontinuous Claudin-1 expression **(O–Q)**, reflecting tight-junction disassembly and structural alteration of epithelial junctions. Scale bars = 50 μm. Data were obtained from 12 Ctrl-Off and 12 Dys-Off mice.

To evaluate the impact of maternal dysbiosis on intestinal structure and enteric neuroanatomy, we performed combined morphometric and immunofluorescence analyses of the large intestine in PND14 offspring (Figure 5). Offspring from dysbiotic dams exhibited reduced body weight compared with control offspring (Figure 5G), suggesting systemic growth delay associated with altered maternal microbiota. Dys-Off intestines displayed thinner crypts (Figure 5H), and reduced muscularis propria thickness in comparison to Ctrl-off (Figure 5I). Immunostaining with the pan-neuronal marker PGP9.5 revealed dense and well-organized enteric networks across the Auerbach’s (myenteric) and Meissner’s (submucosal) plexuses in control offspring (Figures 5A–C), whereas the area of Auerbach plexus (Figure 5J), but not its density (Figure 5K) were significantly reduced in the Dys-Off in comparison to Ctrl-off, indicating impaired epithelial and muscular development. Following ENS assessment, we examined epithelial tight-junction integrity using Claudin-1 immunostaining. In control offspring, Claudin-1 exhibited continuous labeling along the apical epithelial border (Figures 5L–N). Dysbiotic offspring showed fragmented and discontinuous Claudin-1 expression with multiple epithelial gaps and reduced fluorescence intensity (Figures 5O–Q), reflecting tight-junction disassembly and barrier disruption. Collectively, these findings demonstrate that maternal dysbiosis leads to reduced offspring body and intestinal growth, compromised Auerbach’s plexus architecture, and loss of epithelial barrier cohesion, underscoring the dual impact of microbial imbalance on enteric neural organization and mucosal integrity.

### 3.5 Maternal dysbiosis differentially alters GAD67-GFP interneuron morphology in layers II/III SSC and MC of offspring

**FIGURE 6:**
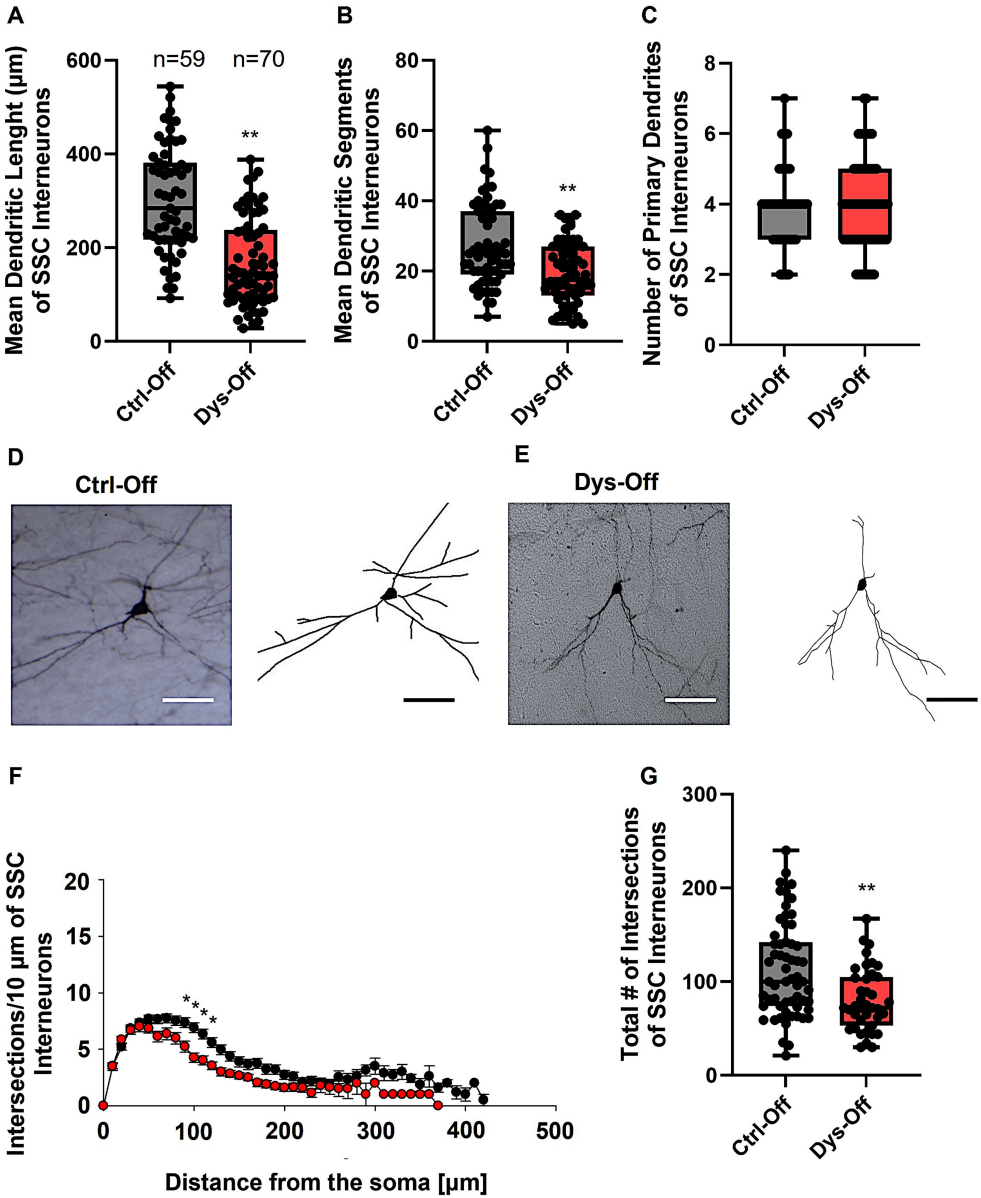
Effect of maternal dysbiosis on the morphology of interneurons in the SSC. **(A)** The box plot in the graph represents the mean dendritic length of interneurons in the control (Ctrl-Off) and dysbiotic (Dys-Off) offspring. **(B)** The box plot in the graph represents the mean values of dendritic segments of interneurons in the Ctrl-Off and Dys-Off groups. **(C)** The box plot in the graph represents the mean values of the total number of primary dendrites of interneurons in the two groups. Representative images of a control interneuron **(D)** and a dysbiotic interneuron **(E)** are presented at 40 × magnification. The corresponding neuronal tracings are shown next to each image. Scale bars: 30 μm. **(G)** Sholl analysis of the control and Dys-Off groups shows a significant decrease in dendritic intersections between 40 and 100 μm from the soma in dysbiotic offspring, using *t*-test post hoc Bonferroni corrections (***P < 0.001). Error bars in **(F)** represent the standard error of the mean (SEM). **(G)** The box plot in the graph represents the total number of dendritic intersections for both groups. For the graphs in **(A–C,G)**, Mann–Whitney U test; **P < 0.01. The number of reconstructed cells per group is provided above the box plot in **(A)**. *N* = 55 reconstructed cells from 8 control mice and *N* = 62 reconstructed cells from 9 dysbiotic mice.

To determine whether maternal dysbiosis impacts inhibitory circuit development in the neocortex, we reconstructed GAD67-GFP–labeled interneurons within the layers II/III of SSC and MC of offspring at PND14. In SSC, control offspring interneurons exhibited highly ramified dendritic arbors with numerous secondary and tertiary branches extending radially from the soma (Figures 6A–E). In contrast, interneurons from dysbiotic offspring showed reduced dendritic length and segments but the primary dendrites were unchanged (Figures 6A–E), indicating impaired postnatal dendritic elaboration. To ascertain in which dendritic compartment the reduction in dendritic complexity occured, we conducted Sholl analyses. The analysis demonstrated a notable decline in dendritic complexity at proximal but not distal dendritic intersections of GAD67-positive interneurons from dysbiotic offspring in comparison to the control group (2-way repeated measures ANOVA, *P < 0.05, Figure 6F). Furthermore, the total number of dendritic intersections of interneuron dendrites was significantly reduced in the dysbiotic offspring (Figure 6G). These data suggested that maternal dysbiosis during gestation results in a reduction in dendritic complexity of proximal dendrites of cortical interneurons in offspring SSC. This morphological simplification reflects deficient maturation of inhibitory networks within the SSC, a region critically involved in sensory processing and cortical excitatory–inhibitory balance.

**FIGURE 7:**
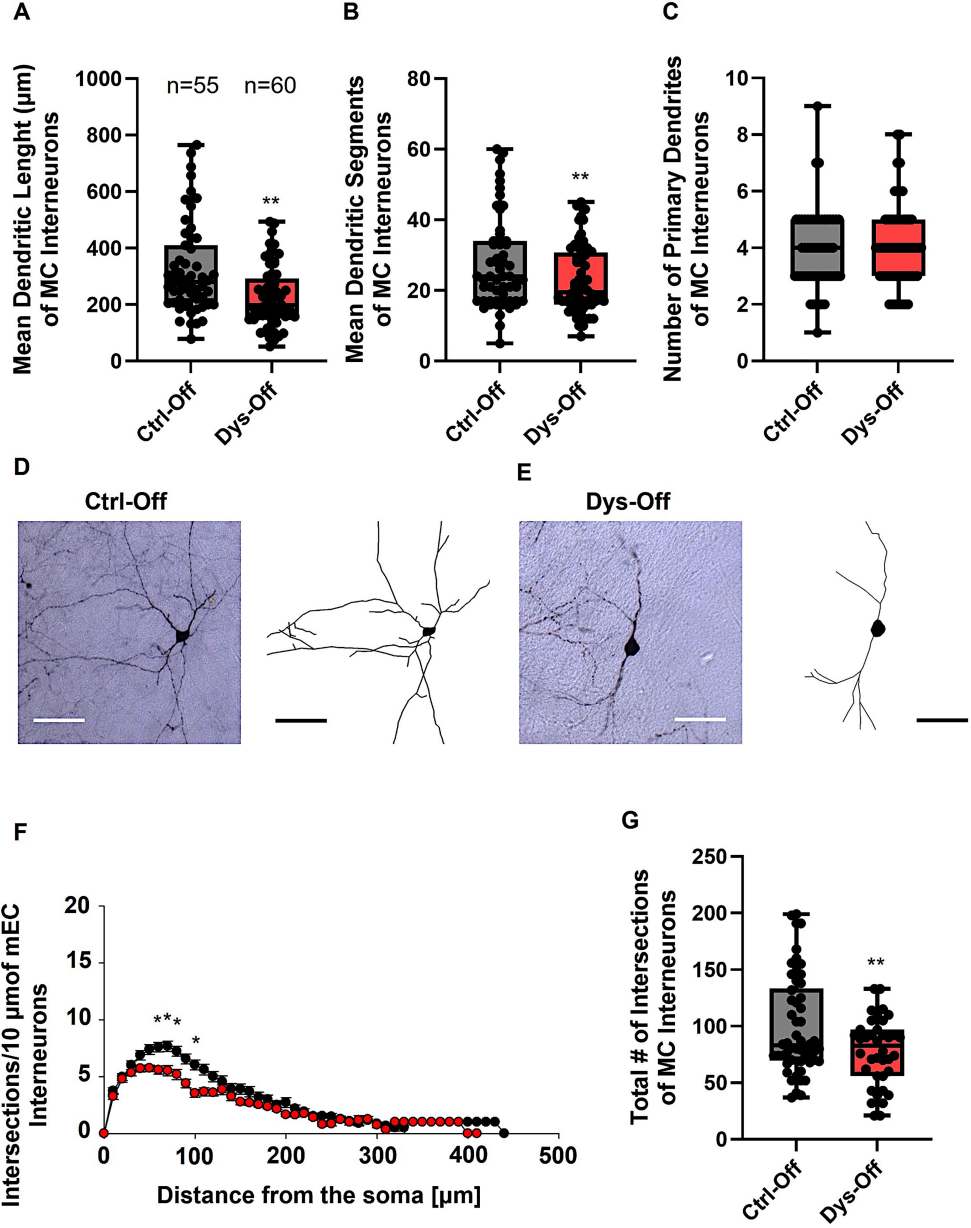
Effect of maternal dysbiosis on the morphology of interneurons in the MC. **(A)** The box plot in the graph represents the mean dendritic length of interneurons in the control (Ctrl-Off) and dysbiotic (Dys-Off) offspring. **(B)** The box plot in the graph represents the mean number of dendritic segments of interneurons in the Ctrl-Off and Dys-Off groups. **(C)** The box plot in the graph represents the mean values of the total number of primary dendrites of interneurons in both groups. Representative images of a control interneuron **(D)** and a dysbiotic interneuron **(E)** are presented at 40 × magnification. The corresponding traced reconstructions are shown next to each image. Scale bars: 30 μm. **(F)** Sholl analysis of the control and Dys-Off groups reveals a significant reduction in dendritic intersections between 40 and 100 μm from the soma in dysbiotic offspring, using *t*-test post hoc Bonferroni corrections (*P < 0.05). Error bars in **(F)** represent the standard error of the mean (SEM). **(G)** The box plot in the graph represents the total number of dendritic intersections for both groups. For the graphs in **(A–C,G)**, Mann–Whitney U test; **P < 0.01. The number of reconstructed cells per group is indicated above the box plot in **(A)**. *N* = 55 reconstructed cells from 8 control mice and *N* = 61 reconstructed cells from 9 dysbiotic mice.

We next evaluated whether similar alterations occur in the MC. In control offspring, MC interneurons displayed the characteristic multipolar dendritic geometry with well-defined primary branches and abundant distal extensions (Figures 7A–E). Offspring from dysbiotic dams, however, exhibited reduced dendritic length and segments, paralleling the phenotype observed in the SSC (Figures 7A–E). To ascertain in which dendritic compartment the reduction in dendritic complexity occured, we conducted Sholl analyses. The analysis demonstrated a notable decline in dendritic complexity at proximal but not distal dendritic intersections of GAD67-positive interneurons from dysbiotic offspring in comparison to the control group (2-way repeated measures ANOVA, *P < 0.05, Figure 7F). Furthermore, the total number of dendritic intersections of interneuron dendrites was significantly reduced in the dysbiotic offspring (Figure 7G). Together, these results demonstrate that maternal dysbiosis induces region-specific cortical interneuron dysmorphogenesis, prominently affecting inhibitory networks in the SSC and MC. The consistent reduction in dendritic architecture across both sensorimotor cortices indicates that microbial imbalance during gestation compromises cortical GABAergic maturation, potentially altering downstream sensorimotor and behavioral integration.

### 3.6 Maternal dysbiosis does not affect interneuron morphology in the layers II/III mEC and CA1 region of the Hp

**FIGURE 8:**
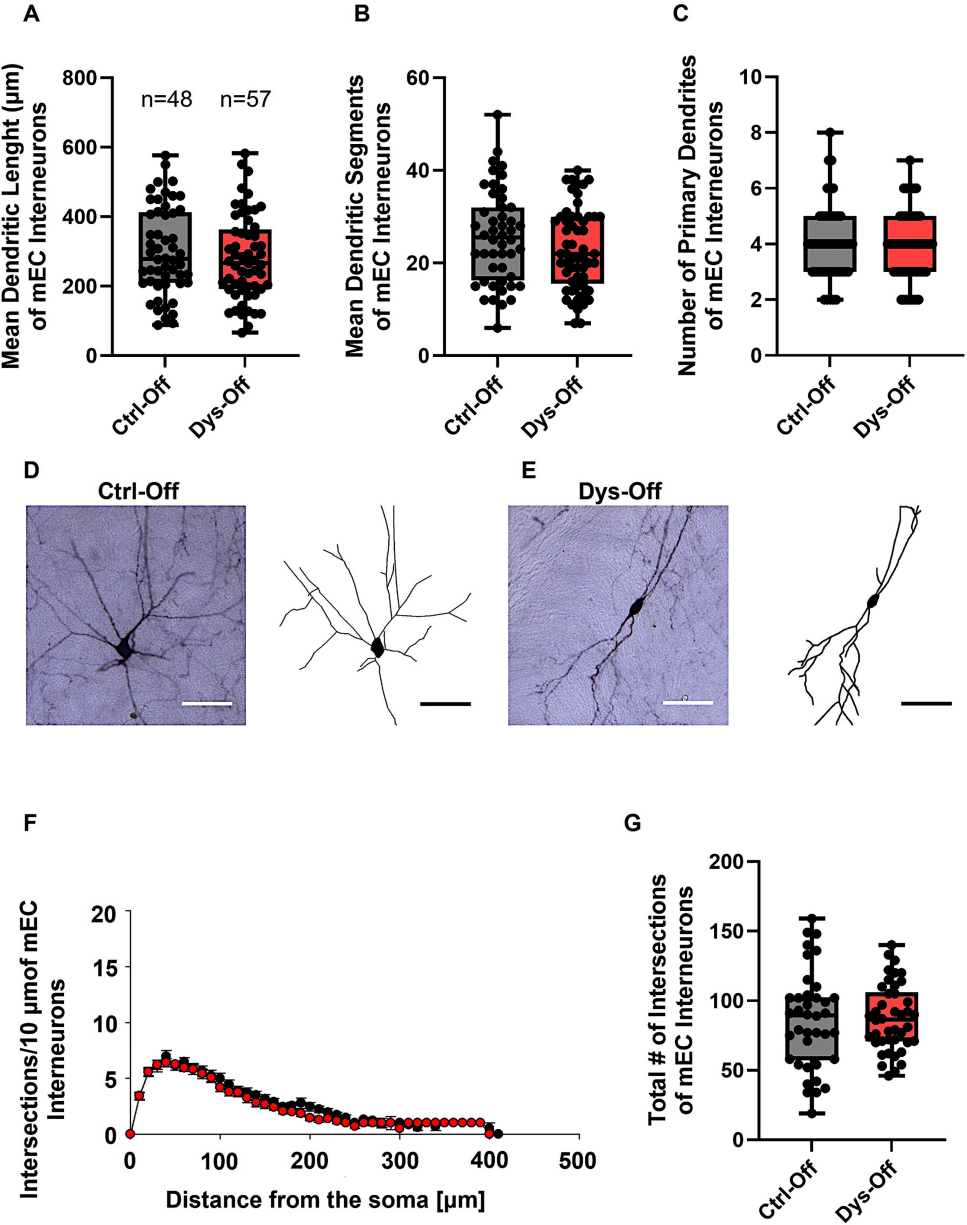
Effect of maternal dysbiosis on the morphology of interneurons in the mEC. **(A)** The box plot in the graph represents the mean dendritic length of interneurons in the control (Ctrl-Off) and dysbiotic (Dys-Off) offspring. **(B)** The box plot in the graph represents the mean number of dendritic segments of interneurons in the Ctrl-Off and Dys-Off groups. **(C)** The box plot in the graph represents the mean values of the total number of primary dendrites of interneurons in both groups. Representative images of a control interneuron **(D)** and a dysbiotic interneuron **(E)** are shown at 40 × magnification. The corresponding traced reconstructions are displayed next to each image. Scale bars: 30 μm. **(F)** Sholl analysis of the control and Dys-Off groups demonstrates no changes. Error bars in **(F)** represent the standard error of the mean (SEM). **(G)** The box plot in the graph represents the total number of dendritic intersections for both groups. The number of reconstructed cells per group is indicated above the box plot in **(A)**. *N* = 48 reconstructed cells from 8 control mice and *N* = 57 reconstructed cells from 9 dysbiotic mice.

**FIGURE 9:**
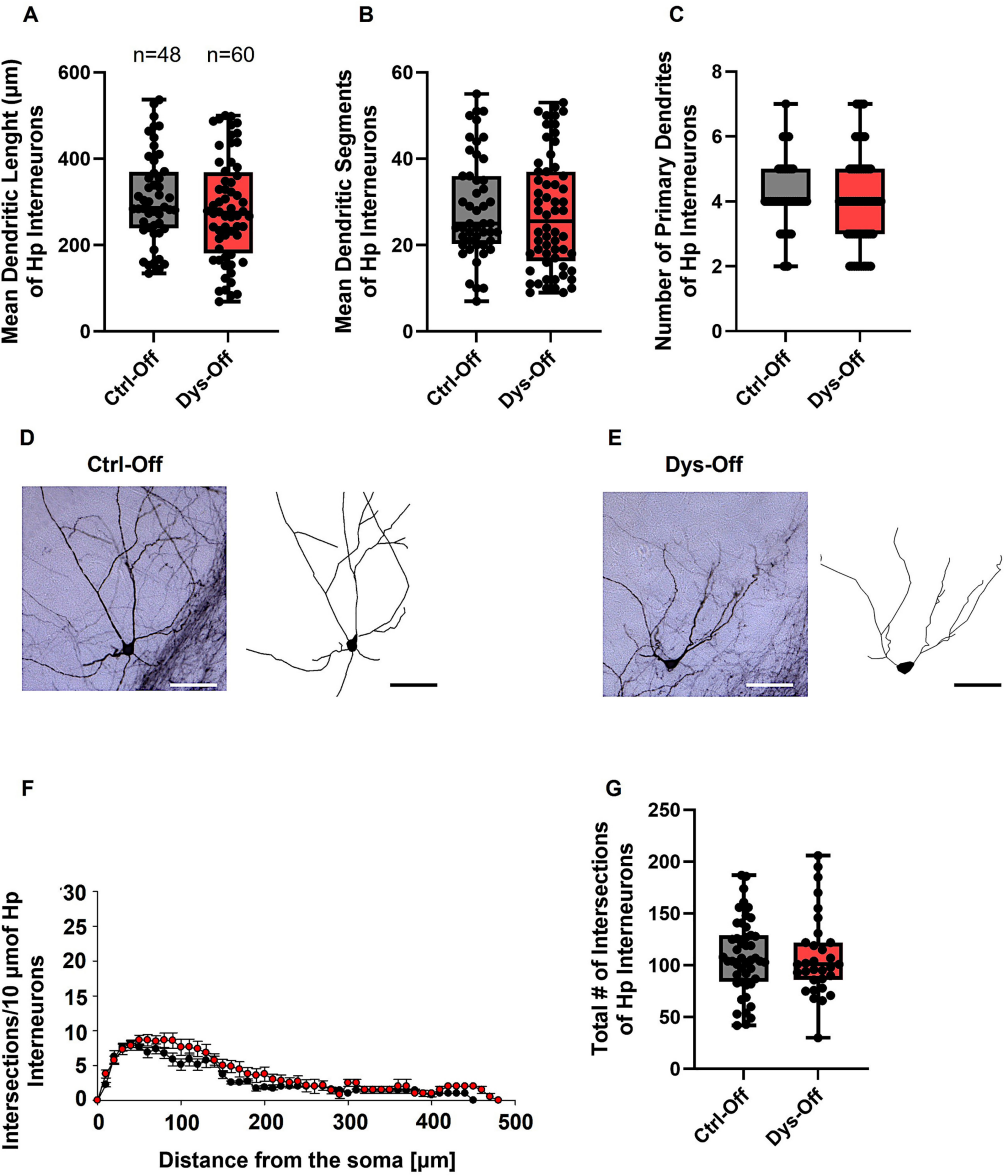
Effect of maternal dysbiosis on the morphology of interneurons in the Hp. **(A)** The box plot in the graph represents the mean dendritic length of interneurons in the control (Ctrl-Off) and dysbiotic (Dys-Off) offspring. **(B)** The box plot in the graph represents the mean number of dendritic segments of interneurons in the Ctrl-Off and Dys-Off groups. **(C)** The box plot in the graph represents the mean values of the total number of primary dendrites of interneurons in both groups. Representative images of a control interneuron **(D)** and a dysbiotic interneuron **(E)** are presented at 40 × magnification. The corresponding neuronal tracings are shown adjacent to each image. Scale bars: 30 μm. **(F)** Sholl analysis comparing control and Dys-Off groups reveals no significant differences in dendritic intersections across distances from the soma. Error bars in **(F)** represent the standard error of the mean (SEM). **(G)** The box plot in the graph represents the total number of dendritic intersections for both groups. The number of reconstructed cells per group is indicated above the box plot in **(A)**. *N* = 48 reconstructed cells from 8 control mice and *N* = 60 reconstructed cells from 9 dysbiotic mice.

To determine whether maternal dysbiosis impacts interneuron structure within limbic regions, we analyzed the morphology of GAD67-GFP–labeled inhibitory interneurons in the layers II/III mEC and CA1 region of the Hp of PND 14 offspring. Interneurons from control (Ctrl-Off) and dysbiotic offspring (Dys-Off) exhibited comparable dendritic arborization length, segments, and branch complexity in mEC (Figures 8A–E) and Hp (Figures 9A–E). Similarly, Sholl analysis showed no change in the number or distribution of dendritic intersections across distances from the soma in mEC (Figure 8F) and Hp (Figure 9F), and total intersection counts remained equivalent in mEC (Figure 8G) and Hp (Figure 9G). These findings indicate that maternal dysbiosis does not influence inhibitory interneuron morphology in the mEC or HP, suggesting that limbic interneurons are structurally resilient to maternal microbial perturbation during early postnatal development.

### 3.7 Maternal dysbiosis reduces GAD67-positive interneuron density in layers II/III MC

**FIGURE 10:**
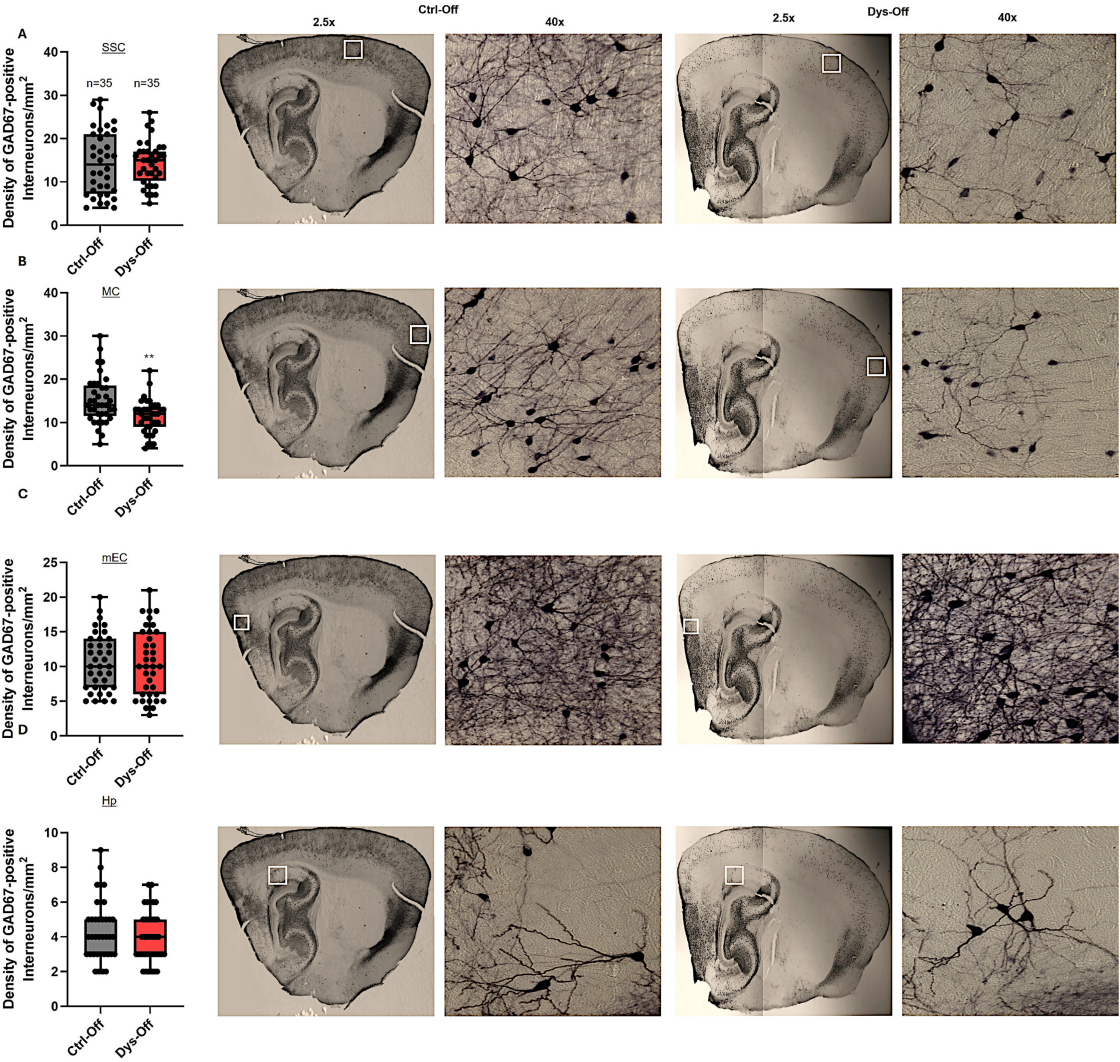
Maternal dysbiosis selectively reduces GAD67-positive interneuron density in the layers II/III of motor cortex of offspring. Quantitative and representative photomicrographs illustrating the effects of maternal dysbiosis on GAD67-positive interneurons in offspring at postnatal day 14 (PND14). Low-magnification images show representative parasagittal sections at comparable rostrocaudal levels. White boxes indicate the regions selected for quantitative analysis. **(A–D)** Box plots on the left show the mean density of GAD67-immunopositive interneurons (cells/mm^2^) in the layers II/III of SSC **(A)**, MC **(B)**, mEC **(C)**, and CA1 region of Hp **(D)** of control offspring (Ctrl-Off, black) and offspring from dysbiotic dams (Dys-Off, red). Data are expressed as mean ± SEM; *p* < 0.05, Mann–Whitney U test. To the right of each graph, representative 4 × low-magnification photomicrographs show the regional distribution of GAD67-positive interneurons within each area, while adjacent 40 × high-magnification images depict individual interneuron morphology within the same field. White boxes on the 4 × images indicate the region enlarged at 40×. Anatomical reference maps from the Allen Brain Atlas (far right) identify the sampled regions, with red boxes marking the analyzed cortical and hippocampal areas. Scale bars: 200 μm (4×), 20 μm (40×). The analyses were performed on 8 control and 9 dysbiotic mice. From each animal, three brain slices were analyzed, and 3 areas of interest (AOIs) were selected per slice.

To determine whether maternal dysbiosis influences the distribution of inhibitory interneurons within cortical and limbic regions, we quantified GAD67-GFP–positive interneuron density across the layers II/III of SSC, MC, mEC, and CA1 region of the Hp of PND14 offspring (Figure 10). Quantitative analysis revealed a significant reduction in GAD67-positive interneuron density in the MC of dysbiotic offspring compared with controls (Figure 10B). In contrast, interneuron density within the SSC, mEC, and Hp remained unchanged between groups (Figures 10A,C,D). These findings suggest that maternal dysbiosis leads to a selective reduction of inhibitory interneuron density in MC, potentially reflecting altered neuronal maturation or survival within specific cortical regions. However, because interneuron density was quantified exclusively in layers II/III, we cannot exclude the possibility that the observed reduction reflects redistribution of interneurons across cortical layers rather than a net loss.

### 3.8 Offspring gut microbial signatures are associated with enteric network and cortical interneuron structural phenotypes

**FIGURE 11:**
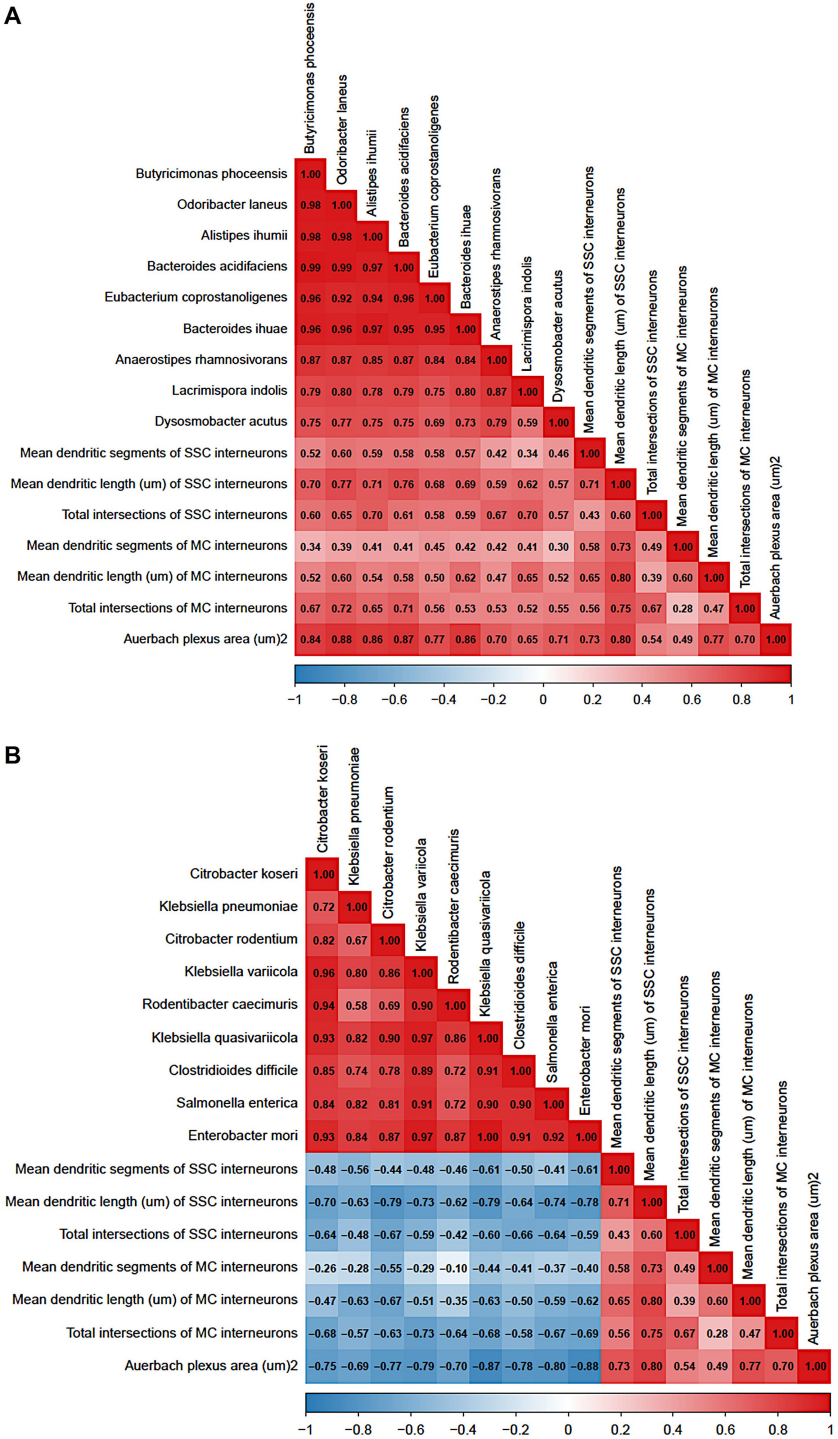
Gut microbial signatures are associated with enteric nervous system architecture and cortical interneuron complexity in offspring. Correlation matrices depict associations between log-transformed bacterial abundance and neuroanatomical parameters measured in offspring (*n* = 24; 12 control and 12 dysbiotic pups). Analyses were restricted to enteric and cortical morphological features that were significantly altered by maternal dysbiosis. **(A)** Health-associated gut commensal taxa show predominantly positive correlations with Auerbach’s plexus area and with dendritic complexity metrics of GAD67-positive interneurons in the somatosensory cortex (SSC) and motor cortex (MC), including mean dendritic length, number of dendritic segments, and total dendritic intersections. **(B)** Dysbiosis-associated pathobionts exhibit inverse correlations with enteric nervous system architecture and cortical interneuron dendritic complexity, while displaying strong positive inter-correlations among pathobiont taxa. Colors indicate Pearson correlation coefficients (red, positive; blue, negative). Correlations were assessed using Pearson’s correlation on log-transformed bacterial abundances.

To assess whether inter-individual variation in gut microbial composition was associated with the enteric and cortical neuroanatomical phenotypes observed in offspring, correlation analyses were performed between bacterial abundance and morphological parameters that were significantly altered by maternal dysbiosis. Analyses were conducted exclusively in pups (*n* = 12; 6 control offspring and 6 dysbiotic offspring). Pearson correlation matrices revealed distinct and opposing association patterns for health-associated commensal taxa and dysbiosis-associated pathobionts (Figure 11). Health-associated commensal bacteria displayed predominantly positive correlations with Auerbach’s plexus area and with dendritic complexity metrics of GAD67-positive interneurons in the somatosensory cortex (SSC) and motor cortex (MC), including mean dendritic length, number of dendritic segments, and total dendritic intersections (Figure 11A). These commensal taxa also exhibited strong positive inter-correlations, consistent with a parallel microbial signature associated with preserved enteric nervous system architecture and cortical inhibitory circuit complexity. In contrast, dysbiosis-associated pathobionts showed widespread inverse correlations with Auerbach’s plexus area and with dendritic complexity measures of SSC and MC interneurons (Figure 11B). Pathobiont taxa were strongly positively correlated with one another, forming a distinct cluster associated with reduced enteric nervous system integrity and simplified cortical interneuron morphology. Together, these findings indicate that gut microbial signatures are closely associated with parallel alterations in enteric and cortical inhibitory circuit architecture, supporting an integrative relationship between microbial composition and neurodevelopmental outcomes in offspring.

## 4 Discussion

### 4.1 Maternal dysbiosis and associated structural changes

This study identifies a strong association between gestational maternal dysbiosis and altered intestinal and cortical development in offspring. Using a validated gestational vancomycin model, we demonstrate that maternal microbial disruption during pregnancy significantly reduces microbial richness, compromises vertical microbial transmission fidelity, and biases inheritance toward pathobiont-enriched communities. These microbial alterations coincide with impaired intestinal barrier integrity, reduced enteric plexus area, and region-specific cortical interneuron dysmorphogenesis in the somatosensory and motor cortices. Collectively, these findings suggest that maternal microbiota integrity may play an important role in structural changes observed in both enteric and central nervous systems in both enteric and central nervous systems during early development. Studies in human showed that perinatal antibiotics diminish vertical transmission of maternal microbes, reducing mother–infant microbial similarity from roughly 70–25% (Ferretti et al., 2018; Jašareviæ et al., 2021). Large infant-cohort metagenomic analyses confirm that early-life antibiotic exposure drives *Enterobacteriaceae* expansion and loss of microbial metabolic diversity (Bokulich et al., 2016). Our results reinforce the concept of dysbiotic inheritance, whereby disruption of maternal microbial continuity causes offspring to acquire microbiota with diminished metabolic and immunomodulatory capacity (Grech et al., 2021). Taxa central to short-chain fatty-acid (SCFA) synthesis, including *Roseburia*, *Faecalibacterium*, and *Blautia*, were underrepresented in dysbiotic dyads, while pro-inflammatory *Gammaproteobacteria* were selectively transmitted. These compositional alterations mirror those reported in mothers with obesity or inflammatory bowel disease, where dysbiosis leads to vertical enrichment of pathobionts and impaired immune tolerance (Gohir et al., 2015). Collectively, these data support the concept that maternal dysbiosis is associated with selective microbial inheritance, reshaping early colonization trajectories and predisposing offspring to inflammation-associated phenotypes. While our regression and similarity analyses suggest altered maternal–offspring microbial continuity, we did not perform cross-fostering or co-housing experiments to directly test vertical transmission mechanisms. Therefore, contributions from cage effects, maternal care behaviors, or changes in milk composition cannot be excluded. Future studies incorporating cross-fostering designs and strain-level microbial tracking will be required to definitively establish causal transmission pathways.

### 4.2 Enteric nervous system alterations

The ENS depends on microbial signaling for neuronal differentiation and circuit maintenance. We observed a marked reduction in the Auerbach’s plexus area and fragmentation of Claudin-1 tight-junction labeling in dysbiotic offspring, indicating altered enteric network organization occurring alongside epithelial structural disorganization. These results align with previous work showing that antibiotic-induced or germ-free conditions delay ENS maturation and disrupt epithelial–neuronal cross-talk (Kabouridis et al., 2015; Obata et al., 2020). Alterations in tight junction organization are frequently associated with dysbiosis-related intestinal inflammation and have been implicated in neurodevelopmental vulnerability through systemic immune modulation (Bonaz et al., 2018). Decreased Claudin-1 continuity reflects structural disassembly of epithelial junction architecture. However, we did not directly assess functional intestinal permeability using *in vivo* tracer assays or electrophysiological measurements. Because systemic inflammatory markers, circulating metabolites, and direct permeability assays were not measured, we cannot determine whether intestinal barrier alterations mediate the cortical phenotypes or represent parallel developmental effects. Therefore, these findings suggest altered barrier organization rather than definitive evidence of increased permeability. The co-occurrence of epithelial structural alteration and reduced ENS plexus area suggests parallel intestinal developmental modulation involving both epithelial and enteric compartments. Microbiota-derived SCFAs act as trophic factors for both epithelial and neuronal cells. Loss of butyrate-producing taxa may contribute to the observed ENS underdevelopment, as butyrate has been shown to promote neuronal excitability and neurogenesis via histone-acetylation-dependent activation of growth genes (Silva et al., 2020). Similar trophic deprivation has been reported in antibiotic-treated neonates, where SCFA supplementation restores enteric neuronal density (De Vadder et al., 2018). Thus, microbial metabolites may represent one potential biochemical conduit linking maternal microbial alterations with offspring ENS maturation.

### 4.3 Cortical interneuron dysmorphogenesis and functional implications

Cortical analysis revealed a reduction in dendritic length and segments of GAD67-positive interneurons within the SSC and MC cortices, while the Hp and mEC were unaffected. This dendritic simplification may reflect disruption of extracellular molecular signaling cascades that guide interneuron arborization during development (Hamad et al., 2023). This pattern demonstrates a clear region-specific vulnerability consistent with differential maturation timelines and metabolic activity across cortical regions. The selective reduction in interneuron density observed in the layers II/III motor cortex further supports the possibility of region-specific developmental vulnerability. Cortical areas mature asynchronously, and interneuron proliferation, tangential migration from the ganglionic eminences, and activity-dependent refinement occur at different rates across cortical territories. Because we did not directly assess progenitor proliferation, migration dynamics, or apoptosis, we cannot determine whether the reduced density in the motor cortex reflects altered interneuron generation, impaired survival, or delayed maturation. Future studies incorporating proliferation markers and lineage-tracing approaches will be required to clarify the developmental mechanisms underlying this regional specificity. Importantly, because our analysis was restricted to layers II/III, the observed reduction in interneuron density may also reflect laminar redistribution rather than an absolute decrease in total interneuron number. Our findings echo those reported in antibiotic-induced adult dysbiosis, where GABAergic interneurons displayed decreased dendritic complexity and density (Nakhal et al., 2025a). It is important to note that the GAD67-GFP mouse line labels the broad population of cortical and hippocampal GABAergic interneurons, encompassing multiple molecular subtypes with distinct morphologies and functional properties (e.g., PV, SST, VIP). The present study therefore reflects population-level structural alterations rather than subtype-specific effects. Because we did not perform additional immunostaining to distinguish interneuron subclasses, we cannot determine whether particular subtypes are selectively vulnerable to maternal dysbiosis. Future investigations employing subtype-specific markers will be required to dissect potential cell-type–specific mechanisms. The current maternal dysbiosis model suggests that early microbial perturbation during gestation is associated with early structural alterations in inhibitory circuitry during a critical developmental window, consistent with prior evidence showing that maternal deprivation leads to persistent alterations in inhibitory interneuron number and morphology within limbic structures such as the amygdala and nucleus accumbens (Aleksic et al., 2023; Nakhal et al., 2025b). Germ-free mice show reduced enteric neuronal density and delayed ganglionic maturation, which normalize after microbial colonization. Hence, maternal dysbiosis, by altering microbial metabolites and intestinal architecture, may be associated with parallel alterations in enteric and cortical development, occurring alongside altered cortical interneuron development in offspring. Several neurological conditions are increasingly recognized as “interneuronopathies” due to their association with inhibitory circuit dysfunction, encompassing disorders such as schizophrenia, bipolar disorder, depression, and epilepsy (Knopp et al., 2008; Leifeld et al., 2022). Even subtle disturbances in the density or dendritic architecture of GABAergic interneurons can disrupt inhibitory control and induce pathological network hyperexcitability, a mechanism implicated in the pathogenesis of epilepsy, schizophrenia, bipolar disorder, and autism spectrum disorders (Ishii et al., 2016; Hu et al., 2017). Maintaining equilibrium between excitatory and inhibitory signaling is essential for stable brain network dynamics. Alterations in GABAergic neurotransmission, whether reduced or excessive, have been linked respectively to epileptiform activity or cognitive dysfunction (Kalueff and Nutt, 1996; Treiman, 2001). Because interneurons govern inhibition within cortical microcircuits, a reduction in GAD67-positive interneuron density may reflect decreased inhibitory tone, rendering these networks more excitable. This interpretation suggests that the loss of inhibitory balance observed because of maternal dysbiosis could lead to broader alterations in cortical and hippocampal network activity. To verify this hypothesis, future studies employing *in vivo* calcium imaging or patch-clamp electrophysiology will be needed to determine whether interneuron loss translates into measurable deficits in inhibitory synaptic transmission. Taken together, the observed decrease in interneuron density in dysbiotic offspring animals supports the premise that gut microbiota disruption may contribute to functional changes in brain circuitry, potentially underlying altered neural excitability in the adult mouse brain.

### 4.4 Immune–metabolic mechanisms and epigenetic regulation

The interplay between maternal dysbiosis and systemic immune activation may further explain the cortical and enteric alterations observed. Antibiotic-induced microbial depletion elevates pro-inflammatory cytokines such as IL-6, IL-17, and TNF-α, which cross the placenta and disrupt GABAergic neuron differentiation via microglial activation (Orchanian and Hsiao, 2025). Simultaneously, the reduction in SCFAs like butyrate and propionate deprives the fetal brain of key histone acetylation signals required for dendritic maturation (Sajdel-Sulkowska, 2023). These converging immune and metabolic insults may contribute to the observed reduction in dendritic complexity, linking maternal gut inflammation to fetal cortical vulnerability. The reduction of Auerbach’s plexus area and Claudin-1 continuity is consistent with altered ENS structure, although functional signaling was not assessed, a critical communication pathway between the gut and the brain. The ENS has been proposed to transmit microbial and metabolic cues via vagal afferents that regulate cortical excitability and interneuron network stability (Foong et al., 2020). Impaired ENS maturation may therefore reduce vagal signaling and alter cortical inhibition. These findings collectively are consistent with hypotheses proposing that the maternal microbiota may influence cortical circuitry through a putative enteric–vagal–cortical signaling axis that warrants functional investigation. Recent evidence indicates that maternal dysbiosis can induce long-term and even transgenerational effects on neurodevelopment through epigenetic remodeling of synaptic and immune-related genes (Liu et al., 2025). Altered DNA methylation patterns in offspring neurons may perpetuate inhibitory circuit abnormalities beyond the first generation. Future experiments using germ-free, cross-fostering, and fecal transplantation models could clarify whether microbial restoration can rescue the observed neural phenotypes.

### 4.5 Model limitations and causality considerations

It is important to note that gestational vancomycin exposure represents a complex maternal perturbation and does not isolate microbiota-specific mechanisms. In addition to altering gut microbial communities, antibiotic treatment may influence maternal immune activation, metabolic homeostasis, bile acid composition, and nutrient absorption, any of which could contribute to offspring phenotypes. Because maternal cytokines, metabolic parameters, and placental inflammatory markers were not directly measured in the present study, we cannot definitively distinguish microbiota-mediated effects from secondary maternal physiological consequences of antibiotic exposure. Future studies incorporating germ-free paradigms, cross-fostering strategies, microbial rescue approaches, and maternal immune profiling will be necessary to establish causality more precisely. Our findings bear important translational implications. Human cohort studies have linked perinatal antibiotic use, cesarean delivery, and maternal dysbiosis to higher incidence of autism, ADHD, and cognitive delays (Alkuwaiti et al., 2025). The GABAergic interneuron network is central to maintaining excitation–inhibition balance; thus, the observed dysmaturation provides a plausible neuroanatomical substrate for such disorders. Targeted interventions, including maternal probiotic or prebiotic therapy, dietary SCFA supplementation, and microbial transplantation, may help restore microbial stability and prevent long-term neurodevelopmental deficits. Importantly, the present study assessed structural parameters and microbial–neuroanatomical correlations but did not directly measure electrophysiological activity, synaptic transmission, or vagal signaling. Therefore, while the observed parallel alterations in enteric and cortical architecture are consistent with gut–brain axis involvement, functional connectivity along this axis remains to be experimentally established.

### 4.6 Summary and outlook

This study primarily used vancomycin, a narrow-spectrum antibiotic that predominantly targets Gram-positive bacteria. Different dysbiosis models, including stress or diet-induced perturbations, may produce distinct microbial and neuroanatomical outcomes. Moreover, while our analysis revealed structural alterations, electrophysiological and behavioral studies will be essential to confirm their functional significance. Future work should integrate metabolomic and transcriptomic approaches to delineate the specific microbial pathways influencing enteric and cortical development. In summary, gestational maternal dysbiosis is associated with altered microbial transmission, barrier integrity, and neuronal development in both intestinal and cortical systems. Whether these early structural alterations persist into adulthood or translate into functional behavioral phenotypes remains to be determined. These findings underscore the intergenerational importance of microbial fidelity in associated with brain and gut developmental processes and highlight the maternal microbiota as a potentially modifiable contributor to neurodevelopmental trajectories of neurodevelopmental health. Importantly, the present study is a descriptive anatomical and microbiome analysis and does not establish causal or functional gut–brain signaling mechanisms, but rather defines structural associations that provide a basis for future mechanistic investigation.

## Data Availability

The datasets presented in this study can be found in online repositories. The names of the repository/repositories and accession number(s) can be found in the article/supplementary material.
